# Portrait of rural emergency departments in Québec and utilization of the provincial emergency department management Guide: cross sectional survey

**DOI:** 10.1186/s12913-015-1242-0

**Published:** 2015-12-23

**Authors:** Richard Fleet, Julien Poitras, Patrick Archambault, Fatoumata Korika Tounkara, Jean-Marc Chauny, Mathieu Ouimet, Josée Gauthier, Gilles Dupuis, Alain Tanguay, Jean-Frédéric Lévesque, Geneviève Simard-Racine, Jeannie Haggerty, France Légaré

**Affiliations:** Department of Family and Emergency Medicine Université Laval, Québec, QC Canada; Research Chair in Emergency Medicine Université Laval, CHAU Hôtel-Dieu de Lévis Hospital, Lévis City, QC Canada; Department of Family and Emergency Medicine, Department of Emergency Medicine, University of Montreal, Hôpital du Sacré-Coeur de Montréal, Montréal, QC Canada; Department of political science, Université Laval, Québec, QC Canada; Direction de l’analyse et de l’évaluation des systèmes de soins et services, Institut national de santé publique du Québec, Université du Québec à Rimouski, Rimouski, QC Canada; Département de psychologie, Université du Québec à Montréal, Montréal, QC Canada; Centre for Primary Health Care and Equity of the University of New South Wales, New South Wales, Australia; Departement of Emergency Medicine, CSSS de la Matapédia, Québec, QC Canada; McGill Research Chair, Family and Community Medicine, Montréal, QC Canada; Department of Family Medicine and Emergency Medicine, Knowledge Transfer and Health Technology Assessment of the CHUQ Research Centre (CRCHUQ), Unité de Recherche Évaluative, Université Laval, Québec, QC Canada; CSSS Alphonse-Desjardins, Research Centre, Hôtel-Dieu de Lévis, 143 Wolfe Street, Lévis, Québec G6V 3Z1 Canada

**Keywords:** Access to care, Rural medicine, Rural emergency medicine, Health services research, Rural critical care, Canadian health care system, Cross sectional survey, Descriptive study, Primary care

## Abstract

**Background:**

Rural emergency departments (EDs) constitute crucial safety nets for the 20 % of Canadians who live in rural areas. Pilot data suggests that the province of Québec appears to provide more comprehensive access to services than do other provinces. A difference that may be attributable to provincial policy/guidelines “the provincial ED management Guide”. The aim of this study was to provide a detailed description of rural EDs in Québec and utilization of the provincial ED management Guide.

**Methods:**

We selected EDs offering 24/7 medical coverage, with hospitalization beds, located in rural or small towns. We collected data via telephone, paper, and online surveys with rural ED/hospital staff. Data were also collected from Québec’s Ministry of Health databases and from Statistics Canada. We computed descriptive statistics, ANOVA and t-tests were used to examine the relationship between ED census, services and inter-facility transfer requirements.

**Results:**

A total of 23 of Québec’s 26 rural EDs (88 %) consented to participate in the study. The mean annual ED visits was 18 813 (Standard Deviation = 6 151). Thirty one percent of ED physicians were recent graduates with fewer than 5 years of experience. Only 6 % had residency training or certification in emergency medicine. Teams have good local access (24/7) to diagnostic equipment such as CT scanner (74 %), intensive unit care (78 %) and general surgical services (78 %), but limited access to other consultants. Sixty one percent of participants have reported good knowledge of the provincial ED management Guide, but only 23 % of them have used the guidelines. Furthermore, more than 40 % of EDs were more than 300 km from levels 1 to 2 trauma centers, and only 30 % had air transport access.

**Conclusions:**

Rural EDs in Québec are staffed by relatively new graduates working as solo physicians in well-resourced and moderately busy (by rural standards) EDs. The provincial ED management Guide may have contributed to this model of service attribution. However, the majority of rural ED staff report limited knowledge or use of the provincial ED management Guide and increased efforts at disseminating this Guide are warranted.

## Background

Providing emergency medical care in rural settings is challenging [1, 2]. Rural populations are generally older, report lower health status and are at a proportionally higher risk of trauma than are urban populations [3–5]. Rural emergency departments (EDs) are important safety nets for the members of the local community. However, over the past 15 years, the limited human and financial resources available in rural Canada have forced efforts to centralize/regionalize health care services in these areas [6–9]. These changes may have reduced timely access to emergency services and, in some cases, decreased the quality of care [10].

For many, Canada’s publically funded universal health care system is a defining feature of this country. Canadian rural citizens may be tempted to look toward Federal legislation, the Canada Health Act, as a safeguard because one of the central components of the legislation is “reasonable access to care” [11]. The intent of accessibility criterion of the Canada Health Act is set to ensure that Canadians “have reasonable access to insured hospital, medical and surgical-dental services on uniform terms and conditions, unprecluded or unimpeded, either directly or indirectly, by charges (user charges or extra-billing) or other means (e.g., discrimination on the basis of age, health status or financial circumstances)” [12]. However, actual provision of health care is under provincial jurisdiction and interprovincial variations are possible without justification.

In 1997, the Canadian Association of Emergency Physicians (CAEP) responded to service cuts with a position paper on rural emergency care [13], listing priority research questions to be addressed. Despite this call for research, surprisingly few studies have been conducted since 1997, and gaps in the knowledge about rural emergency services still preclude evidence-based decisions about allocation of resources and services. Furthermore, the extent and the nature of services currently provided in rural EDs remains unclear.

According to our pilot studies [14], the province of Québec seems to provide more comprehensive local access to services in rural EDs than that offered in other parts of Canada [15]. For example, less than 30 % of rural EDs in other provinces have 24/7 access to a local CT scanner, ICU or general surgeon. The reasons for the discrepancies are not clear, but one hypothesis is that this may be attributable to a published provincial policy/guidelines and for emergency care in Québec [16]. The provincial ED management Guide defines the expected support services in EDs as per hospital designation, and includes specific sections pertaining to rural EDs [16]. Hospital designation is determined by several factors, including ED census. The provincial ED management Guide was developed in 2000 and revised in 2006, with the objective of making all stakeholders accountable for quality of care in EDs. It is unclear whether or not Québec has been successful in implementing the provincial ED management Guide recommendations and if implemented guidelines have resulted in increased access to quality care.

A major research initiative is underway in Québec, with the objective of providing a detailed portrait of rural EDs and a better understanding of the use/impact of the provincial ED management Guide on quality of care [17]. As an initial step, this article presents a detailed description of Québec’s rural EDs and the use of the provincial ED management Guide.

## Methods

The protocol for the larger multi-center assessment program was approved by the research ethics committee at the Alphonse–Desjardins Center for Health and Social Services (CSSS AD). Informed consent to participate in the study was not obtained from participants, because data were from hospital database. Anonymity was respected at all levels of the procedure.

Methodology details are presented in the published protocol [17]. The Québec study was conducted in several phases. This paper reports data obtained in phase 1 of the study (described below). This project is a descriptive and evaluative study of rural EDs in Québec. The EDs included in the study offered 24/7 medical coverage, had hospitalization beds and were located in a “rural or small towns”. The Statistics Canada definition of “rural or small towns” is the following: “the population living in towns and municipalities outside the commuting zone of larger urban centers (i.e., outside the commuting zone of centers with a population of 10,000 or more)” [18]. The 26 Québec rural EDs were identified using the Health Canada Establishment Guide and confirmed by the Québec Ministry of Health and Social Services and the “*Direction Nationale des Urgences”*.

### Data collection

To develop a comprehensive portrait of all rural EDs in Québec in 2010, data were collected via telephone, paper, and online surveys with rural ED/hospital staff, from Québec’s Ministry of Health databases, and from Statistics Canada. Briefly, the telephone, paper, and online surveys were used to obtain data concerning (1) hospital center characteristics (e.g., referral centres, availability of local intensive care unit [ICU] beds); (2) availability of health information technology (e.g., Internet and wireless Internet access); (3) knowledge transfer activities (e.g., quality assurance, Journal club); (4) ED variables (e.g., triage level, wait time, average hospital stay, on number of annual visits, number of stretchers and number of transfers between facilities) were gathered in conjunction with MSSS “*Direction Nationale des Urgences*”, (5) availability of 24/7 diagnostic services (e.g., laboratory, basic radiography, Computerized Tomography (CT) Scanner, Magnetic Resonance Imaging (MRI), ultrasound, portable ultrasound); (6) medical and paramedical staff (e.g., number of emergency doctors, years of experience and level of training, percentage of locum doctors per period, availability of specialists, number and level of training of nurses, presence of other health professionals). Prior to the study, this list of variables was submitted for review to a group of experts in emergency medicine and trauma as well as to decision-makers and leaders of professional associations including clinicians, the heads of professional associations, the Québec college of physicians), policymakers, and the senior management of medical schools [19]. All sociodemographic variables were obtained from Statistics Canada website.

### Statistical analyses

Data were entered and verified by two research assistants and analyzed with SAS 9.3 (SAS Institute Inc, Cary, North Carolina, USA). The analyses were conducted in collaboration with an independent biostatistics service from the Statistical Consulting Service of Université Laval. Means, medians, and percentages were calculated for variables. We used ANOVAs and *t* tests to examine the relationships between available services, ED census and inter-facility transfer requirements.

## Results

The overall ED participation rate was 88 % (23/26 EDs). The “rural or small towns” where the EDs were located had a mean population of 5 889 (Standard deviation [SD] = 4 064), and a population density of 133 per km^2^ (SD = 167). The median age was 49 years and 9 % of the members of the local community were over the age of 75 years. The general characteristics of the rural EDs and rural hospitals are presented in Table 1. Briefly, rural hospitals had an average of 43 acute care beds (SD = 25) and 6 ED stretchers. EDs received a mean of 18 813 annual patient visits (SD = 6151). Seventy seven percent of visits were for low-acuity conditions (Level 4 or Level 5 triage). Average wait times (time from patient registration or triage to the time to see a physician) were just under three hours and the average length of stay was roughly 12 h. Mean length of stay was 15 h for hospitalized patients (awaiting beds), and 11 h for non-hospitalized patients. Forty four percent of EDs are more than 300 km away from a level 1 trauma center and 48 % are more than 300 km from a level 2 trauma center, and only 30 % of these EDs had air transport access.

**Table 1:** General characteristics of hospital and ED performance

Characteristics	Means ± SD^a^ or %
No of hospital beds	43 ± 25
No of stretchers	6 ± 3
Annual ED patient visits	18 813 ± 6 151
Triage level	
Level 1	1 %
Level 2	2 %
Level 3	20 %
Level 4	36 %
Level 5	42 %
ED Performance (wait in hours)	
ED length of stay	12 ± 4
Wait time	3 ± 2
Average length of stay in the ED for hospitalized patients	15 ± 7
Average length of stay in the ED for non-hospitalized patients	11 ± 3
Percentage of ED stays over 48 h	2.2 %
Distance to trauma center level 1 or 2	
EDs > 300 km from a Level 1 trauma center	43 % (10/23)
EDs > 300 km from a Level 2 trauma center	48 % (11/23)
Air medevac capability	30 % (7/23)

^a^Standard deviation

Table 2 presents descriptive statistics of ED staff. Almost all EDs were staffed by a solo physician. Full-time and part-time physicians constituted 46 and 54 % of ED physicians, respectively. Most physicians had fewer than 10 years of experience. Only 6 % had Emergency medicine residency training or certification (e.g., Certificate of the College of Family Physicians of Canada - Emergency Medicine (CCFP-EM) or Fellow of the Royal College of Physicians (FRCP) in emergency medicine. On average, physicians worked alongside three nurses on shift. A respiratory technician was on call 24/7.

**Table 2:** Staffing of the Québec’s 23 Rural EDS

Characteristic	Means ± SD^a^ or %
Physicians Working full time in ED	6 ± 7
Physicians Working part time in ED	7 ± 6
Years of experience	
0–5 years	31 %
6–10 years	20 %
11–15 years	18 %
16–20 years	18 %
Over 20 years	12 %
Percentage of locum doctors per period	
January to April	14 %
May to August	15 %
September to December	13 %
No of nurses in ED	
Day shift	3 ± 1
Evening shift	3 ± 1
Night shift	2 ± 1
Nurses certification	
Auxiliary	1 ± 3
Québec’s College (CEGEP)	15 ± 8
Bachelors	4 ± 2

^a^Standard error

As presented in Table 3, the majority of Québec’s rural EDs had local access to basic laboratory services, x-ray services and advanced imagery services. Moreover, most EDs had in-house 24/7 access to an ICU (78 %), a general surgeon (78 %), and an anaesthetist (65 %). Fewer than 20 % of EDs had access to a paediatrician or orthopaedist. All EDs have access to Internet, whereas 39 % have access to telemedicine support.

**Table 3:** Access to 24/7 support services and consultants in Québec’s rural EDs

Support services	
Laboratory	100 % (*n* = 23/23)
Basic x-ray services	91 % (*n* = 21/23)
ICU	78 % (*n* = 18/23)
Portable ultrasound machine	78 % (*n* = 18/23)
CT scanner	74 % (*n* = 17/23)
Formal ultrasound (radiologist)	30 % (*n* = 7/23)
Access to Telehealth	39 % (9/23)
Consultant available 24/7	
Surgeon	78 % (*n* = 18/23)
Respiratory therapist	73 % (*n* = 16/22) ^a^
Anesthetist	65 % (*n* = 15/23)
Psychiatrist	48 % (*n* = 11/23)
Internist	39 % (*n* = 9/23)
Obstetrician/gynecologist	35 % (*n* = 8/23)
Orthopedist	17 % (*n* = 4/23)
Pediatrician	13 % (*n* = 3/23)
Neurologist	0 % (*n* = 0/23)

^a^One missing value

Higher volume EDs (more than 20 000 annual visits) did not require significantly more inter-facility transfers than low volume EDs (less than 15 000 annual visits) (*p* = 0.681). More transfers were required in EDs that did not have an ICU versus those that did (*p* = 0.02). A higher need for *urgent* inter-facility transfer was significantly associated with *not* having 24/7 access to a pediatrician or an orthopedic surgeon (*p* < 0.05) (Table 4).

**Table 4:** Relationship between 24/7access to services, ED volume and inter-facility transfers

Variables	Overall transfers, means ± SD	*p* value	Emergency transfers, means ± SD	*p* value	Nonemergency transfers, means ± SD	*p* value
ICU						
Yes	270 ± 127	0.022	44 ± 29	0.082	226 ± 114	0.048
No	382 ± 69		85 ± 34		297 ± 44	
CT scanner						
Yes	293 ± 116	0.929	54 ± 8	0.839	239 ± 103	0.854
No	299 ± 159		50 ± 45		250 ± 125	
Portable ultrasound machine						
Yes	321 ± 118	0.059	60 ± 35	0.055	261 ± 104	0.088
No	198 ± 105		27 ± 27		171 ± 87	
Ultrasound (radiologist)						
Yes	304 ± 130	0.8130	52 ± 28	0.960	252 ± 113	0.771
No	290 ± 126		53 ± 39		237 ± 107	
Psychiatrist						
Yes	328 ± 119	0.223	58 ± 39	0.521	270 ± 108	0.225
No	263 ± 127		48 ± 33		215 ± 102	
Obstetrician/gynecologist						
Yes	278 ± 138	0.678	36 ± 25	0.067	242 ± 120	0.986
No	303 ± 121		63 ± 38		241 ± 102	
Surgeon						
Yes	301 ± 159	0.694	58 ± 37	0.104	243 ± 101	0.920
No	269 ± 118		33 ± 24		236 ± 135	
Internist						
Yes	300 ± 141	0.863	38 ± 25	0.094	262 ± 125	0.503
No	290 ± 119		62 ± 39		228 ± 95	
Pediatrician						
Yes	299 ± 101	0.941	32 ± 10	0.032	267 ± 95	0.661
No	294 ± 130		56 ± 37		238 ± 109	
Orthopedist						
Yes	280 ± 90	0.763	30 ± 9	0.009	251 ± 85	0.834
No	297 ± 133		58 ± 37		240 ± 112	
Anesthetist						
Yes	289 ± 121	0.787	57 ± 39	0.378	231 ± 105	0.556
No	305 ± 138		44 ± 30		261 ± 113	
ED Volume						
Low (<15 000)	287 ± 153	0.681^£^	50 ± 46	0.602^£^	237 ± 121	0.788^*^
Medium (15 00–19 999)	260 ± 117		42 ± 33		218 ± 95	
High (≥20 000)	317 ± 120		60 ± 33		257 ± 111	

^*^ *P* value from anova test

This study asked ED chairs and chief nurses whether or not they knew about or used the ED Guide. Sixty one percent of participants have reported good knowledge of the provincial ED management Guide, but only 23 % of them have used the guidelines (Table 5).

**Table 5:** Knowledge and utilization of the provincial ED management Guide *n* = 62

Knowledge of the provincial ED management Guide	
None	6,5 % (4/62)
A little	32,3 % (20/62)
Moderately	41,9 % (26/62)
A lot	19,4 % (12/62)
Utilization of the provincial ED management Guide	
Never	24,2 % (15/62)
Sometime	53,2 % (33 /62)
Often	19,4 % (12/62)
Always	3,2 % (2/62)

## Discussion

Few studies have examined rural emergency care in Canada. As a first step, a detailed descriptive study of rural EDs is required in order to further contribute to evidence-based resource allocation and planning. This study collected data on almost 90 % of Québec’s rural EDs. Overall; these EDs treat more than 400 000 patients annually. In contrast to our preliminary data from other provinces in Canada, Québec’s rural EDs have high patient volumes and appear to offer more 24/7 local support services [14, 15]. This study hypothesized that service attribution may reflect provincial policy and could be attributed in part to the existence of the provincial ED management Guide. Yet, surprisingly, emergency healthcare professionals reported limited knowledge and use of the provincial ED management Guide. Nevertheless, in absence of standards in rural emergency care, Québec’s unique policy of providing comprehensive 24/7 local access resources such as CT scanner, surgical and critical care services may be judicious in the context that rural EDs are distant from tertiary trauma centers and have limited air medevac capabilities.

### ED characteristics, staffing and performance

Québec rural EDs receive a significant number of ED visits relative to the size of the local population. In fact, rural EDs in Québec receive, on average more than 40 % more consultations than do rural EDs in other provinces of Canada [14]. These consultations are, for the most part, for lower-acuity conditions. Given the limited access to family doctors in rural communities in Québec, it is possible that patients depend on EDs for their acute care needs [20]. In Québec, 21 % of citizens do not have a family physician (and access to one takes an average wait time of 466 days) [21] compared to 15 % in the rest of Canada [22, 23]. Having a primary care doctor does not guarantee access for emergent consultation. In fact, 45 % of Canadians that have a family doctor cannot obtain same-day consultations [22]. Thus EDs will likely remain a safety net for minor emergencies in rural areas. Considering reasonable wait times reported here this may not be a dramatic outcome [20].

Despite the high volume of consultations, wait times in Québec rural EDs are within or approximate national guidelines that were recently published by CAEP [24]. These guidelines suggest that wait times should be under two hours and total time spent in the ED be less than 8 h [24]. Interestingly, a recent report by the Canadian Institute for Health Information (CIHI) shows that national ED wait times average 9.2 h [25]. CIHI did not report rural versus urban wait times. Noteworthy is that Canada has one of the worst ED wait times in developed countries [22].

EDs are staffed by a combination of family doctors who exclusively practice full-time emergency medicine, and family doctors who also have other duties. A total of 14 % of shifts require locum coverage (back-up doctors). Québec has a back-up system consisting of a list of volunteer physicians that staff EDs as necessary called “mécanisme de dépannage”. This system was established primarily to maintain 24/7 ED coverage in small communities. In 2010, a total of 287 physicians across the province were identified as back-ups, many at the service of the hospitals that participated in this study (240 physicians). One report indicated over 13,000 shifts required locum coverage in 2010. In that year alone, the program cost ten million dollars for physicians’ transportation and lodging [26].

Moreover, in order to improve access to emergency care in rural areas, the Québec government developed a program that favours placement of graduating doctors in non-urban, rural, or regional areas. In their first 3 years of practice, new graduates are encouraged to practice in these areas with various financial incentives that can reach upwards of 40 % more remuneration than urban doctors for the same medical billing code [27]. Also, the regional plans of medical staffing in family medicine authorize, for every administrative region of Québec, a target number for family doctors’ recruitment, which allows a fair distribution of new family physicians. Without these programs, the problem of access to physicians in rural centers could be far worse.

Given this context, our finding that 31 % of doctors working in rural areas have fewer than 5 years of experience is not surprising. However, it is unclear how many young physicians stay in rural communities and for how long. A few studies have examined retention in rural areas and multiple factors affect this aspect [28–30]. However, no study has examined recruitment and retention in the context of rural emergency practice *per se*. In light of current and foreseen staffing shortages in emergency medicine, future studies on this aspect are required [31]. A subsequent phase of this study will address this.

The practice of rural emergency medicine is stressful, and may by particularly so for a young physician working with limited consulting and support services. Solutions to the disparity between rural and urban services are complex and multifaceted. Doctors in rural practice become vulnerable to burnout owing to the high workload and low level of collegial and consultant support [28]. Data collected in our pre-study phase suggested that access to resources (e.g., CT scanner) and consultants were among the most important issues for ED physicians [32]. The fact that the vast majority of rural EDs in Québec have 24/7 access to a CT scanner, general surgeon, and an ICU may reflect this need and justify why this is part of the provincial ED management Guide.

### Trauma care

Trauma is the 5th leading cause of mortality nationwide [33] and the first cause in patients under the age of 40 [34]. Over the last 40 years, trauma care has improved dramatically and the mortality rate among victims with serious injuries (ISS > 12) fell from 52 % in 1992 to 8,6 % in 2002 in Québec [35]. This dramatic change is believed to be attributable to prevention and the organization of trauma systems. The timely care of patients in level 1 and level 2 trauma centers has also contributed to this excellent result. While 77.5 % of Canadians have access to Level 1 and level 2 trauma centers within a conservative “golden two hours”, marked geographic disparities in access persist [36]. Access to trauma centers is critical because the risk of trauma is three times higher in rural than in urban patients, and the risk of trauma death is twice higher [37]. This study demonstrates that 44 and 48 % of Québec’s rural EDs are respectively, over 300 km from of the level 1 and level 2 trauma centers. Given this distance, it is highly unlikely that trauma patients would reach trauma centers within the recommended time frame. Québec has no helicopter transport and 16 rural hospitals do not have airplane medevac access. Initial management of patients in Québec rural hospitals is therefore highly probable, if not the norm. We are presently conducting a detailed study to specifically address the issue of rural trauma care in Québec. In particular, the study will address the impact of local resources on trauma care.

### Inter-facility transfers

Inter-facility transfers imply that the local center is not able to provide appropriate care for the specific reason transfer is requested. Despite good access to resources in Québec’s rural EDs in comparison to rural EDs elsewhere in Canada, access to life- and limb-saving consultations remains limited. For example, rural hospitals in Québec do not have access to cardiac catheterization, or to sub-surgical specialties such as orthopedics, neurosurgery and plastics. In our study, fewer than 40 % have access to an internist, 13 % to a pediatrician and 0 % to neurology. Moreover, the present study demonstrated that approximately 294 inter-facility transfers (data not shown) per year are required with 18 % on an urgent basis. This finding is consistent with the only other report on inter-facility transfer requirements in a rural setting in Canada [38]. Rourke et al*.* showed that 1.6 % of patients require transfer, most commonly for: orthopedic care (24 %), CT scanner (14 %), and pediatric consultation (8,7 %) [38].

Inter-facility transfers lead to reduced ambulance coverage in rural areas posing a risk to communities. Ambulance transfer is in itself a risky high speed transport, with ambulance crashes occurring with greater frequency and severity than crashes involving vehicles of similar size and weight characteristics [39]. Moreover, results indicate that while there is a greater incidence in urban ambulance accidents, the percentage of ambulance crashes with injuries and the severity of the injuries is greater in the rural settings [39–41]. Future national studies must relate inter-facility transfers to locally available equipment. Québec has a complex EMS system comprising of 50 private companies. This appears to be unique feature where most provinces have provincially managed systems. A subsequent phase of this study will address prehospital care in detail.

Thus providing access to services in rural areas, the province of Québec is a unique and possible forward thinking policy. Future studies are required to evaluate whether this model of care is cost-effective, safe and could favor recruitment and retention of physicians.

### The provincial emergency department management Guide

The provincial ED management Guide is, to our knowledge, the most recent and concise available document that was designed to make all stakeholders accountable in the process of care of emergency patients. It specifies the services that should be accessible in the province’s EDs based on the number of annual visits to the department and other variables. The Guide also has a section dedicated to rural emergency departments [16].

To our knowledge, Québec is the only Canadian province to have published a comprehensive Guide. For the purpose of the current paper, we focused solely on exploring whether or not participants were aware of the Guide, and whether or not they perceived it to be useful. The large proportion of respondents who were not aware that the Guide existed was a surprise. The provincial ED management Guide is not new, the first version was published in 2000 and a revised version was published in 2006. It is possible that new graduates and employees were not exposed to this tool, and that knowledge transfer concerning the provincial management guide was not sustained over the years. A new version of the guide is presently being developed, and the information in this study may be useful in planning knowledge transfer strategies. An extension of this research will be to conduct a detailed study of the barriers/facilitators to the use of the Guide and better understand its contribution to the level of care and resources offered in rural settings.

In absence of evidence-based standards in care, written policy may serve as a guideline for emergency care. Certainly, the fact that most rural EDs have 24/7 access to a CT scanner, general surgical services and ICU is unique in Canada and could reflect this policy. In absence of standards of care in rural EDs and awaiting data, policy that aims at providing access to services may be a cautious approach to safe care for vulnerable rural populations.

### Is the Québec model better?

Providing 24/7 surgical, anesthetic and ICU care carries a cost. Decision makers across Canada have centralized these services on the basis of regrouping expertise, facilitating management and obviously reducing costs. The Québec model thus appears dramatically different from elsewhere in Canada, at least with respect to our preliminary data [14]. The salient question remains what system is more cost-efficient and safe? In terms of cost, a recent report by CIHI suggests *per capita* annual healthcare spending in Québec is one of the lowest [42]. This same report states that Québec provides among the best value per dollar spent among Canadian provinces [42]. Yet, this report does not address issues of quality of emergency care *per se*. Future studies need to examine the relationship between the level of resources/services locally offered in rural hospitals and emergency specific quality of care indicators.

In Canada, a series of priority emergency care sensitive indicators have been developed by Schull et al*.*, [43] They include: ED Operations, Patient Safety, Main Management, Pediatrics, Cardiac, Respiratory, stroke, Sepsis/Infection [43]. They are currently being validated mainly in urban academic centers. The indicators have not been operationalized for rural settings. Thus, it may be possible that quality of care indicators cannot be easily captured in rural settings with limited computerized data/patients records. A subsequent phase of this study will address this issue. While awaiting results, in the context of preliminary reports from elsewhere in Canada and the US that suggest increased mortality from trauma, stroke, in rural versus urban hospitals, caution must be exercised in service attribution decisions [44–46].

### Limitations

The present study constitutes a detailed descriptive portrait of rural EDs in Québec. For the purpose of conducting a nationwide study on rural EDs, this work focused on EDs providing 24/7 physician coverage located in “Rural small town” communities in hospitals with acute care hospitalization beds. The study did not examine access to the full scope of emergency services in Québec; it excluded facilities such as Centre Local de Services Communautaires (CLSCs), “Groupe de médecin de famille (GMF)”, nursing stations and private clinics, which provide basic emergency care to thousands of patients per year. However, the vast majority of these facilities provide only daytime services. They have limited resources (ex. CT scanners and specialists) and are unlikely to provide the full scope of emergency care for life or limb threatening disease. Furthermore this study did not report data concerning outcomes and costs associated with level of access to services.

This study did not collect data on rural ED service areas, and only reported local municipality populations. Determining hospital service areas is a complex and unreliable task. Hospitals may provide specific or specialty services such as imaging and dialysis for rural areas. Furthermore, patients may seek emergency treatment at the center of their choice, even if the chosen facility is not the closest one to their home. It is therefore quite likely that rural hospitals serve territories and populations that are considerably larger than the local population figures reported here.

## Conclusion

Rural EDs in Québec are staffed by relatively new graduates working as solo physicians in well-resourced and moderately busy (by rural standards) EDs. The EDs are isolated from trauma centers and have limited access to air transport. The Provincial ED Management Guide may have contributed to this model of service attribution by advocating for local services rather than inter-facility transport. However, the majority of rural ED staff report limited knowledge or use of the Québec Guidelines and increased efforts at disseminating this are warranted. Stakeholders nationwide may also want to examine Quebec’s unique model of rural ED service attribution.
